# Extra-esophageal symptoms in individuals with and without erosive esophagitis: a case–control study in Albania

**DOI:** 10.1186/s12876-021-01658-z

**Published:** 2021-02-16

**Authors:** Edite Sadiku, Eqerem Hasani, Indrit Këlliçi, Iris Mone, Fatjona Kraja, Bledar Kraja, Genc Burazeri

**Affiliations:** 1 University Clinic of Gastrohepatology and Hepatology Service, University Hospital Center “Mother Teresa”, Dibra Street 371, 1001 Tirana, Albania; 2 Emergency Departments, University Hospital Center Mother Teresa, Dibra Street 371, Tirana, Albania; 3 Division of Physiology, Department of Biomedical and Experimental Sciences, Faculty of Medicine, University of Medicine, Dibra Street 371, Tirana, Albania; 4 Endoscopy Unit, Service of Surgery, Regional Hospital Durrës, Telat Noga Street, Durrës, Albania; 5 Department of Laboratory, University Hospital Center Mother Teresa, Dibra Street 371, Tirana, Albania; 6 University Clinic of Oncology, University Hospital Center Mother Teresa, Dibra Street 371, Tirana, Albania; 7 Department of International Health, School for Public Health and Primary Care (CAPHRI), Faculty of Health, Medicine and Life Sciences, Maastricht University, 6200 MD Maastricht, The Netherlands

**Keywords:** Albania, Erosive reflux esophagitis, Extra-esophageal symptoms, GERD

## Abstract

**Introduction:**

Erosive reflux esophagitis caused a large clinical spectrum of symptoms. Our aim was to assess the prevalence of extra-esophageal symptoms in individuals with and those without erosive esophagitis in Albania.

**Methods:**

A case–control study was conducted at the Regional Hospital of Durres, the second main district in Albania, a transitional country in South Eastern Europe, including 248 patients with erosive esophagitis (aged 46.5 ± 16.3 years) and 273 controls (aged 46.4 ± 16.0 years; response rate: 70%) enrolled during the period January 2013–June 2014. Both cases and controls underwent upper endoscopy. Information on socio-demographic characteristics and lifestyle factors was also collected. Binary logistic regression was used to assess the association of erosive esophagitis and extra-esophageal symptoms.

**Results:**

Patients with erosive esophagitis had a higher prevalence of excessive alcohol consumption, smoking, sedentarity, non-Mediterranean diet and obesity compared to their control counterparts (9% vs. 5%, 70% vs. 49%, 31% vs. 17%, 61% vs. 49% and 22% vs. 9%, respectively). Upon adjustment for all socio-demographic characteristics and lifestyle/behavioral factors, there was evidence of a strong association of erosive esophagitis with chronic cough (OR = 3.2, 95% CI = 1.7–5.8), and even more so with laryngeal disorders (OR = 4.4, 95% CI = 2.6–7.5). In all models, the association of erosive esophagitis with any extra-esophageal symptoms was strong and mainly consistent with each of the symptoms separately (fully-adjusted model: OR = 4.6, 95% CI = 2.9–7.3).

**Conclusion:**

Our findings indicate that the prevalence of extra-esophageal symptoms is higher among patients with erosive esophagitis in a transitional country characterized conventionally by employment of a Mediterranean diet.

## Introduction

Erosive reflux esophagitis is the most common esophageal complication of gastroesophageal reflux disease (GERD) causing a large clinical spectrum of symptoms. Furthermore, the reflux of gastric contents into the esophageal lumen may involve not only the esophageal mucosa, but may also damage the extra-esophageal tissues such as oropharynx, larynx, and respiratory tract. It is well-known that heartburn and acid regurgitation are typical esophageal symptoms of GERD [1], whereas common extra-esophageal manifestations include throat clearing, sore throat, chronic cough and asthma [1–3]. Although several epidemiological studies from Western countries have shown a significant association between GERD and chronic cough, chronic laryngitis, and asthma, the prevalence of extra-esophageal symptoms in patients with GERD vary widely by populations [4–6]. Also, patterns of symptoms [type and severity] differ between cases with or without erosive esophagitis, whereas the frequency of symptoms correlates with the amount of esophageal acid exposure [7, 8]. Conversely, there are insufficient data on the clinical characteristics of GERD in patients with laryngeal or respiratory symptoms [9, 10].

In Albania, a developing Southeast European country, epidemiological studies have been mainly based on the general population focusing on the prevalence and lifestyle correlates of the typical symptoms of GERD [11, 12]. The available evidence indicates a population prevalence of heartburn and acid regurgitation, which are typical symptoms of GERD, of 12%, and smoking, physical inactivity, and obesity but not alcohol consumption as determinants of GERD in the Albanian adult population [11]. Furthermore, dietary factors, such as consumption of fried foods alone have been linked to an increased risk of typical symptoms of GERD, whereas employment of a predominantly Mediterranean diet has been related to a decreased risk of GERD in a representative population-based sample of Albanian adults [11, 12].

There has been conducted one endoscopy-based study comparing the prevalence of esophageal and extra-esophageal symptoms in a group of Albanian patients with GERD diagnosed by endoscopy [13]. Also, several clinical-based studies from Western countries have investigated the prevalence of extra-esophageal symptoms in various degrees of reflux erosive esophagitis [4, 14, 15]. However, the independent factors related to the development extra-esophageal manifestations remain unclear.

In this framework, the main objective of our study was to assess the prevalence of extra-esophageal symptoms in individuals with [cases] and those without [controls] erosive esophagitis.

## Methods

### Study design

This was a case–control study conducted at the Regional Hospital of Durres, the second main district in Albania after Tirana, the capital. Two hundred and forty-eight cases and 273 controls were enrolled in the study during the period 07 January 2013–30 June 2014. Controls were selected from Durres catchment areas.

### Study population

Cases consisted of consecutive new patients aged 18–70 years who presented with typical and/or atypical reflux symptoms and who were diagnosed with erosive reflux esophagitis by upper gastrointestinal endoscopy during 07 January 2013–30 June 2014 (N = 248). The erosive reflux esophagitis was graded according to the Los Angeles (LA) classification criteria based on the extent of visible erosions: grade A, one or more mucosal breaks no longer than 5 mm, none of which extends between the tops of the mucosal folds; grade B, one or more mucosal breaks more than 5 mm long, none of which extends between the tops of the mucosal folds; grade C, mucosal breaks that extend between the tops of two or more mucosal folds, but which involve less than 75% of the esophageal circumference; and grade D, mucosal breaks which involve at least 75% of the esophageal circumference. The exclusion criteria for cases were as follows: (1) previous GERD; (2) Barrett’s esophagus; (3) history of gastrointestinal surgery and/or gastrointestinal malignancies; (4) taking daily anticholinergics or prokinetics drugs; (5) using non-steroid or steroid anti-inflammatory drugs; (6) pregnancy; (7) history of asthma, heart or pulmonary diseases (treated with ACE inhibitors), and; (8) severe diseases of other organs such as severe liver or kidney diseases, or other severe systemic conditions. Subjects who had received any acid-suppressive drugs within the last four weeks before endoscopy were also excluded. Patients were further divided into two groups according to the severity of esophagitis: LA grade A/B: mild erosive esophagitis, and severe erosive esophagitis: LA grade C/D. Hiatal hernia was recorded as the occurrence of the Z line more than 2 cm above the cardio-esophageal junction.

During the same time period (07 January 2013–30 June 2014), two controls for each case were targeted for recruitment among family members (siblings and/or cousins) of the patients at Durres Hospital (overall N = 496). Inclusion criteria (based on interview and medical history) consisted of individuals aged 18–70 years with no history of previous reflux diseases or use of medications against such diseases. Of the 496 targeted individuals, 51 did not meet the inclusion criteria. Furthermore, individuals unable to provide a clear medical history or who reported typical reflux symptoms more than once per week were excluded (N = 48). In addition, 115 individuals refused to participate. No significant differences in socio-demographic characteristics were found between participants and non-participants in the control group. All the remaining controls (N = 282) with no reflux symptoms agreed to undergo an upper endoscopy examination. Those with endoscopic findings related to GERD or hiatal hernia (N = 9) were also excluded from the study. The final sample included 273 controls (overall response rate: 273/496 = 55%; response rate among eligible controls: 273/388 = 70%).

### Data collection

A physician (medical doctor) completed a standard questionnaire to cases and controls at the time of endoscopy. The questionnaire was designed to collect data on participants’ health conditions, socio-demographic characteristics and lifestyle habits [11, 12]. The socio-demographic data included age, sex, place of residence (urban vs. rural areas), marital status (dichotomized in the analysis into: married vs. not married), educational level (trichotomized into: low, middle and high), employment status (dichotomized into: employed/retired vs. unemployed) and income level (dichotomized into: low vs. average/high). The lifestyle factors included current smoking status (yes vs. no), alcohol consumption (< 1drink/week, 1–6 drinks/week and ≥ 1 drink/day), dietary type (based on frequency of consumption of four main food items: traditional dishes, fruit and vegetables, olive oil, and fish [each item assessed in a scale ranging from ‘frequent’ to ‘no consumption’]; a summary score was calculated or each participant which in the analysis was dichotomized into: Mediterranean diet versus non-Mediterranean diet) [12] and physical activity (low, moderate and high). In addition, height and weight were measured, based on which body mass index (BMI) was calculated for each participant (and subsequently trichotomized in the analysis into: normal weight, overweight and obesity).

All participants were asked to identify the presence of typical and/or extraesophageal symptoms of GERD. The typical gastroesophageal symptoms of GERD were defined as presence of heartburn or acid regurgitation. Heartburn was defined as a retrosternal burning sensation [1]. Acid regurgitation was defined as the perception of flow of refluxed gastric content into the mouth or throat [1]. Participants were also asked to self-assess individually the severity of their typical symptoms which were divided into three categories: no/mild, moderate, or severe. The extra-esophageal symptoms of GERD were defined as chronic cough, throat clearing, sore throat and globus sensation. Chronic cough was defined as a cough that persists eight weeks or longer without having lung disorders. Throat clearing was defined as an instinctive attempt to remove an irritant in the throat. Sore throat was defined as a pain, scratchiness or irritation of the throat that often worsens when swallowing without bacterial or viral infection. Globus sensation was defined as the persistent feeling of a lump in the throat when not swallowing. In the analysis, throat clearing, sore throat and globus sensation were grouped into ‘laryngeal disorders’.

The study was approved by the Department of Biomedical Sciences of the Faculty of Medicine, Tirana, Albania and all participants gave written informed consent after being explained the aim and procedures of the study.

### Statistical analysis

The statistical review of the study was performed by a biomedical statistician.

Student’s t-test was used to compare the distribution of age between cases with erosive esophagitis and controls. Conversely, Fisher’s exact test was used to compare the distribution of the other socio-demographic characteristics and lifestyle factors between cases with erosive esophagitis and controls. Similarly, Fisher’s exact test was employed to compare the distribution of typical gastroesophageal symptoms between cases with mild erosive esophagitis and those with severe erosive esophagitis.

Binary logistic regression was used to assess the association between erosive esophagitis (outcome variable) and extra-esophageal symptoms (independent variables alias “predictors”). More specifically, the three predictor variables included any extra-esophageal symptoms, chronic cough and laryngeal disorders. Crude (unadjusted) logistic regression models were initially run. Next, age-adjusted models were conducted. Subsequently, logistic regression models were adjusted for all socio-demographic characteristics of study participants (age, sex, marital status, residence, education, employment status and income). Finally, logistic regression models were additionally adjusted for lifestyle/behavioral factors (smoking, alcohol consumption, physical activity, BMI and dietary score). Odds ratios (ORs), their 95% confidence intervals (95% CIs) and *P* values were calculated for all the logistic regression models. Hosmer–Lemeshow goodness-of-fit test was used to assess the validity of the logistic regression models.

In all cases, a *P* value ≤ 0.05 was considered as statistically significant.

The statistical analysis was conducted in SPSS (Statistical Package for Social Sciences, version 17.0).

## Results

Mean age in patients with erosive esophagitis was similar to the control group (46.5 ± 16.3 years vs. 46.4 ± 16.0 years, respectively) (Table 1). About 34% of cases and 40% of controls resided in rural areas. About 72% of cases and 76% of controls were currently married. A high educational attainment was slightly more prevalent in controls than in cases (22% vs. 19%, respectively). The proportion of unemployed individuals was higher in cases compared to the control group (36% vs. 23%, respectively, *P* < 0.01). Patients with erosive esophagitis had a higher prevalence of excessive alcohol consumption and especially smoking compared to their control counterparts (9% vs. 6% and 70% vs. 49%, respectively, both *P* < 0.01). Furthermore, cases had a higher prevalence of non-Mediterranean diet than the control group (60% vs. 49%, respectively, *P* < 0.01). A sedentary lifestyle was also more prevalent in cases than in controls (31% vs. 17%, respectively, *P* < 0.01). The prevalence of obesity was considerably higher in cases than in the control group (22% vs. 9%, respectively, *P* < 0.01) (Table 1).

**Table 1:** Distribution of socio-demographic characteristics and lifestyle factors in Albanian patients with erosive esophagitis and in the control group

Variable	Erosive esophagitis (N = 248)			Controls (N = 273)	*P*^†^
	Mild erosive esophagitis (grade A/B) (N = 230)	Severe erosive esophagitis (grade C/D) (N = 18)	Total (N = 248)		
Age (years) (mean ± SD)	46.6 ± 16.4	44.4 ± 15.5	46.5 ± 16.3	46.4 ± 16.0	0.952
*Place of residence*					
Urban areas	149 (64.8)^**a**^	14 (77.8)	163 (65.7)	163 (59.7)	
Rural areas	81 (35.2)	4 (22.2)	85 (34.3)	110 (40.3)	
*Marital status*					
Single/divorced/widowed	65 (28.3)	5 (27.8)	70 (28.2)	65 (23.8)	0.271
Married	165 (71.7)	13 (72.2)	178 (71.8)	208 (76.2)	
*Educational level*					
Low	83 (36.1)	6 (33.3)	89 (35.9)	94 (34.4)	0.760
Middle	107 (46.5)	4 (22.2)	111 (44.8)	119 (43.6)	
High	40 (17.4)	8 (44.4)	48 (19.4)	60 (22.0)	
*Employment status*					
Employed/pension	148 (64.3)	11 (61.1)	159 (64.1)	211 (77.3)	0.001
Unemployed	82 (35.7)	7 (38.9)	89 (35.9)	62 (22.7)	
*Income level*					
Low	139 (60.4)	4 (22.2)	143 (57.7)	105 (38.5)	< 0.001
Average-high	91 (39.6)	14 (77.8)	105 (42.3)	168 (61.5)	
*Current smoker*					
No	69 (30.0)	6 (33.3)	75 (30.2)	139 (50.9)	< 0.001
Yes	161 (70.0)	12 (66.7)	173 (69.8)	134 (49.1)	
*Alcohol consumption*					
< 1 drink/week	131 (57.0)	11 (61.1)	142 (57.3)	191 (70.0)	0.009
1–6 drinks/week	79 (34.3)	4 (22.2)	83 (33.5)	67 (24.5)	
≥ 1 drink/day	20 (8.7)	3 (16.7)	23 (9.3)	15 (5.5)	
*Physical activity*					
Low	72 (31.3)	4 (22.2)	76 (30.6)	47 (17.2)	< 0.001
Moderate	144 (62.6)	11 (61.1)	155 (62.5)	148 (54.2)	
High	14 (6.1)	3 (16.7)	17 (6.9)	78 (28.6)	
*BMI*					
Normal weight (BMI ≤ 25)	18 (7.9)	8 (44.4)	26 (10.5)	69 (25.3)	< 0.001
Overweight (BMI: 25.1–29.9)	158 (69.0)	9 (50.0)	167 (67.6)	180 (65.9)	
Obese (BMI ≥ 30)	53 (23.1)	1 (5.6)	54 (21.9)	24 (8.8)	
*Dietary type*					
Non-Mediterranean diet	139 (60.4)	12 (66.7)	151 (60.9)	133 (48.7)	0.003
Mediterranean diet	91 (39.6)	6 (33.3)	97 (39.1)	140 (51.3)	

^**†**^Comparison between all cases with erosive esophagitis (N = 248) and controls (N = 273). *P* values from chi-square test, except the age which was compared by use of student’s t-test

^**a**^Absolute numbers and column percentages (in parentheses)

The prevalence of hiatal hernia and severe heartburn were higher in cases with severe erosive esophagitis than in those with mild erosive esophagitis (44% vs. 21%, respectively, *P* = 0.03 and 83% vs. 50%, respectively, *P* = 0.03) (Table 2). On the other hand, the prevalence of gastric-duodenal ulcer and severe regurgitation were similar in the two groups (11% vs. 13%, respectively and 78% vs. 70%, respectively).

**Table 2:** Distribution of typical gastroesophageal symptoms among patients with erosive esophagitis

Variable	Mild erosive esophagitis (grade A/B) (N = 230)	Severe erosive esophagitis (grade C/D) (N = 18)	Total (N = 248)	P*
*Heartburn*				
No/mild	41 (17.8)	1 (5.6)	42 (16.9)	0.027
Moderate	73 (31.7)	2 (11.1)	75 (30.2)	
Severe	116 (50.4)	15 (83.3)	131 (52.8)	
*Regurgitation*				
No/mild	23 (10.0)	1 (5.6)	24 (9.7)	0.763
Moderate	45 (19.6)	3 (16.7)	48 (19.4)	
Severe	162 (70.4)	14 (77.8)	176 (71.0)	
*Hiatal hernia*				
No	182 (79.1)	10 (55.6)	192 (77.4)	0.028
Yes	48 (20.9)	8 (44.4)	56 (22.6)	
*Gastric-duodenal ulcer*				
No	201 (87.4)	16 (88.9)	217 (87.5)	0.604
Yes	29 (12.6)	2 (11.1)	31 (12.5)	

^*^P values from Fisher’s exact test

Table 3 presents the association of extra-esophageal symptoms with erosive esophagitis. In crude (unadjusted) models (model 1), there was a strong positive association between erosive esophagitis and chronic cough (OR = 2.8, 95% CI = 1.6–4.6), or laryngeal disorders (OR = 4.4, 95% CI = 2.8–6.9). The associations were not affected upon age-adjustment (model 2). After additional control for all the other socio-demographic characteristics (model 3), the relationship with chronic cough did not change, whereas the association with laryngeal disorders was slightly accentuated (OR = 2.7, 95% CI = 1.6–4.6 and OR = 4.6, 95% CI = 2.9–7.5, respectively). Upon further adjustment for lifestyle/behavioral factors (model 4), the association of erosive esophagitis with chronic cough was strengthened (OR = 3.2, 95% CI = 1.7–5.8), whereas the relationship with laryngeal disorders was slightly weakened (OR = 4.4, 95% CI = 2.6–7.5). In all models, the association of erosive esophagitis with any extra-esophageal symptoms was strong and mainly consistent with each of the symptoms separately (fully-adjusted model: OR = 4.6, 95% CI = 2.9–7.3) (Table 3, model 4).

**Table 3:** Association of extra-esophageal symptoms with erosive esophagitis—odds ratios (OR: erosive esophagitis cases vs. controls) and 95% confidence intervals (95% CIs) from binary logistic regression

Variable	Cases (N = 248)	Controls (N = 273)	Model							
			Model 1^b^		Model 2^c^		Model 3^d^		Model 4^e^	
			OR	95% CI	OR	95% CI	OR	95% CI	OR	95% CI
*Any extra-esophageal symptoms*										
No	124 (50.0)^**a**^	222 (81.3)	1.00	Reference	1.00	Reference	1.00	Reference	1.00	Reference
Yes	124 (50.0)	51 (18.7)	4.35	2.94–6.45	4.34	2.93–6.46	4.14	2.93–6.65	4.58	2.86–7.33
*Chronic cough*										
No	196 (79.0)	249 (91.2)	1.00	Reference	1.00	Reference	1.00	Reference	1.00	Reference
Yes	52 (21.0)	24 (8.8)	2.75	1.64–4.62	2.74	1.63–4.63	2.69	1.57–4.61	3.18	1.75–5.81
*Laryngeal disorders*										
No	159 (64.1)	242 (88.6)	1.00	Reference	1.00	Reference	1.00	Reference	1.00	Reference
Yes	89 (35.9)	31 (11.4)	4.38	2.78–6.88	4.37	2.77–6.89	4.64	2.88–7.46	4.37	2.55–7.48

^**a**^Absolute numbers and column percentages (in parentheses)

^b^Crude (unadjusted) models

^c^Age-adjusted models

^d^Adjusted for socio-demographic characteristics (age, sex, marital status, residence, education, employment status and income)

^e^Adjusted for socio-demographic characteristics and lifestyle/behavioral factors (smoking, alcohol consumption, physical activity, BMI and dietary type (Mediterranean diet vs. non-Mediterranean diet))

## Discussion

In this case–control study we assessed the prevalence of extra-esophageal symptoms among individuals with and without erosive reflux esophagitis in Albania. We found that 50% of the patients with erosive reflux esophagitis had at least one of the extra-esophageal symptoms. We also demonstrated a significant association between extra-esophageal symptoms [chronic cough and laryngeal disorders] and erosive reflux esophagitis.

Our findings are similar to previous studies reporting a significant association between erosive reflux esophagitis and extra-esophageal symptoms [4, 9, 10, 16, 17]. Within the ProGERD study, about 35% of the patients with erosive reflux disease had extra-esophageal symptoms [4], whereas Raiha et al. [18] found extra-esophageal symptoms in 57% of elderly patients with erosive esophagitis. GERD is the third leading cause of chronic cough accounting for 20% of the cases [19]. In the present study, chronic cough was significantly associated with erosive esophagitis in individuals undergoing upper gastrointestinal endoscopy. This association is supported by previous studies which have suggested a significant relationship between chronic cough and erosive esophagitis [4, 20]. We also found that laryngeal disorders were the most frequent extra-esophageal symptoms, occurring in 36% of the patients with erosive esophagitis disclosed by upper gastrointestinal endoscopy. Therefore, the upper gastrointestinal endoscopy should be part of the evaluation in patients with suspected reflux-related chronic cough and laryngeal disorders [17, 21, 22]. However, different results have been reported from using upper gastrointestinal endoscopy in patients with laryngeal symptoms. A population-based case–control study showed increased odds ratios for laryngeal symptoms [OR = 2.0] in cases with esophagitis compared with controls [23], Oh et al. [9] reported that only 21% of all patients with laryngeal symptoms had erosive esophagitis, whereas a study in the UK did not identify a significantly increased risk of laryngeal disorders following a diagnosis of GERD [24]. Differences in diagnostic procedures upon hospitalization may explain, at least to some extent, differences in the results between various studies [23]. In any case, further research is needed on this topic.

Despite the evidence of several epidemiological and clinical studies that GERD is associated with various extra-esophageal symptoms [1], the pathogenesis of symptom manifestations remains unclear. To date, two possible explanations for the extra-esophageal symptoms are considered: the direct noxious effects of reflux acid on pharyngeal and laryngeal mucosal surface and the indirect activation of vagally-mediated reflex through stimulation of sensory nerves from the contact with refluxed gastric content into the distal esophagus [3]. Furthermore, it has been suggested that erosive reflux esophagitis patients are more susceptible to supra-esophageal reflux [21]. In a case control-study, Savarino et al. [25] reported a higher proportion of reflux episodes reaching the proximal esophagus in patients with erosive esophagitis, compared with a control group. Also, previous studies have found that the numbers of volume and acid reflux episodes are important in reflux-esophageal lesions [26]. Animal studies have reported that the combination of gastric content and bile acids is very injurious to the larynx, whereas human studies demonstrated that acid exposure reaching proximal esophagus is significantly increased in patients with laryngeal disorders [27, 28]. However, the presence of extra-esophageal symptoms of GERD in the absence of endoscopic signs of esophageal mucosal break suggests that factors other than acid reflux, such as esophageal mucosal sensitivity, abnormal esophageal contraction, and psychological factors may cause extra-esophageal reflux symptoms [29, 30].

This study investigated for the first time the prevalence of extra-esophageal symptoms in individuals with and without erosive reflux esophagitis in Albania, a Mediterranean country in South Eastern Europe. Other strengths of our study were the recruitment of consecutive new patients and the use of endoscopy also among the controls without symptoms who agreed to participate, in line with similar approaches used elsewhere [30–34]. Furthermore, the upper endoscopy was conducted by one experienced endoscopist and the assessment of symptoms was done by one physician, which avoids the possibility of interobserver variability among endoscopists/physicians. However, the majority of cases included in our study had mild degrees of GERD (grades A and B) which according to the Lyon Consensus [35] would have required a more precise diagnosis by means of functional tests which were not conducted in our study. Also, endoscopy has a low sensitivity in GERD diagnosis [35]. We were able to adjust for major recognized lifestyle factors which increase the risk of extra-esophageal symptoms. Also, the exclusion of patients with asthma, patients treated with cough-inducing medications and prior PPI intake reduces the concern that our results may be biased by unrecognized reflux [36, 37]. Despite its contribution, we are aware of potential limitations of the current study. The study limitations include the relatively small sample size, the recruitment procedure from one hospital only, and the data collection which was conducted more than 6 years ago. Furthermore, findings from case–control studies are not assumed to be causal.

In conclusion, we showed that the prevalence of extra-esophageal symptoms was higher among patients with erosive reflux esophagitis in a transitional country characterized conventionally by employment of a Mediterranean diet. In the future, prospective clinical studies in multiple medical centers are needed in order to generalize our findings in the overall Albanian population and beyond. Further studies are needed to investigate the cause of different extra-esophageal symptoms in different categories of GERD patients. In particular, as the majority of GERD patients pertain to the NERD population without erosive esophagitis, future studies should compare the rate of extra-esophageal symptoms between NERD and erosive patients.

## Data Availability

The datasets used and/or analysed during the current study are available from the corresponding author on reasonable request.

## Abbreviations

GERD: Gastroesophageal reflux disease
LA classification: Los Angeles classification
BMI: Body mass index
